# Examining the Feasibility of Quantifying Receptor Availability Using Cross-Modality Paired-Agent Imaging

**DOI:** 10.1007/s11307-021-01629-6

**Published:** 2021-07-20

**Authors:** Boyu Meng, Negar Sadeghipour, Margaret R. Folaron, Rendall R. Strawbridge, Kimberley S. Samkoe, Kenneth M. Tichauer, Scott C. Davis

**Affiliations:** 1 Thayer School of Engineering, Dartmouth College, 03755 Hanover, NH USA; 2 Biomedical Engineering, Illinois Institute of Technology, Chicago, IL 60616 USA; 3 Geisel School of Medicine, Dartmouth College, 03755 Hanover, NH USA

**Keywords:** Receptor availability, Molecular imaging, Paired-agent imaging, Drug-target engagement, Fluorescence tomography, MRI, Contrast agent, Pharmacokinetics, Tumor

## Abstract

**Purpose:**

The ability to noninvasively quantify receptor availability (RA) in solid tumors is an aspirational goal of molecular imaging, often challenged by the influence of non-specific accumulation of the contrast agent. Paired-agent imaging (PAI) techniques aim to compensate for this effect by imaging the kinetics of a targeted agent and an untargeted isotype, often simultaneously, and comparing the kinetics of the two agents to estimate RA. This is usually accomplished using two spectrally distinct fluorescent agents, limiting the technique to superficial tissues and/or preclinical applications. Applying the approach in humans using conventional imaging modalities is generally infeasible since most modalities are unable to routinely image multiple agents simultaneously. We examine the ability of PAI to be implemented in a cross-modality paradigm, in which the targeted and untargeted agent kinetics are imaged with different modalities and used to recover receptor availability.

**Procedures:**

Eighteen mice bearing orthotopic brain tumors were administered a solution containing three contrast agents: (1) a fluorescent agent targeted to epidermal growth factor receptor (EGFR), (2) an untargeted fluorescent isotype, and (3) a gadolinium-based contrast agent (GBCA) for MRI imaging. The kinetics of all three agents were imaged for 1 h after administration using an MRI-coupled fluorescence tomography system. Paired-agent receptor availability was computed using (1) the conventional all-optical approach using the targeted and untargeted optical agent images and (2) the cross-modality approach using the targeted optical and untargeted MRI-GBCA images. Receptor availability estimates between the two methods were compared.

**Results:**

Receptor availability values using the cross-modality approach were highly correlated to the conventional, single-modality approach (*r* = 0.94; *p* < 0.00001).

**Conclusion:**

These results suggest that cross-modality paired-agent imaging for quantifying receptor availability is feasible. Ultimately, cross-modality paired-agent imaging could facilitate rapid, noninvasive receptor availability quantification in humans using hybrid clinical imaging modalities.

**Supplementary Information:**

The online version contains supplementary material available at 10.1007/s11307-021-01629-6.

## Introduction

Over the past several years, therapeutics designed to target specific molecular features of individual tumors, such as the expression of cell-surface receptors, have become an increasingly central feature of clinical cancer care and new drug development. In this context, the ability to noninvasively quantify the cell-surface receptor availability (RA) for guiding personalized treatment in solid tumors is a central aim of molecular imaging. This capability could be used to supplement anatomical diagnostic information, quantify the availability of drug targets, assess drug-target engagement, and aid in preclinical development of new therapeutics [1–3]. For example, this information could be used to establish an appropriate therapeutic regimen and longitudinally monitor both the evolution of RA over time and whether the receptor-targeted drug is engaging its target as expected. Unfortunately, noninvasive quantitation of RA in solid tumors using molecular imaging approaches with a single receptor-targeted contrast agent is often intractable due to the persistence of nonspecific contrast agent accumulation in the tumor and variability in blood flow among and within tumors [4–6]. To address this challenge, paired-agent imaging (PAI) strategies have been developed to compensate for the confounding effects of nonspecific accumulation and delivery [7, 8]. The general PAI framework involves imaging the dynamic behavior of a pair of contrast agents (often co-administered): one targeted to the receptor of interest and the other a non-targeted isotype [8, 9]. RA can be estimated by either fitting a kinetic model to time-activity curves or applying ratio-based mathematical operations, distinguishing the specific agent-receptor signal from the confounding non-specific accumulation and delivery effects exhibited by the targeted agent [8–11].

To date, PAI has been developed and validated primarily using optical imaging of two spectrally distinct fluorescently labeled agents, largely due to the modality’s capacity to separate multiple colocalized agents with different fluorescent labels [10]. Optical imaging is exceptionally well-suited to image superficial tissues, such as exposed tumors in preclinical models or fluorescence-guided surgery applications in humans, and we have previously reported on using fluorescence tomography–based PAI to estimate RA and drug-receptor engagement in subsurface tumor xenografts in mice [10–13]. Yet, even with sophisticated tomographic techniques, the high scattering behavior of visible and near-visible photons in tissue limits optical PAI applicability in human tissue volumes greater than a few centimeters in diameter.

Deploying the PAI technique using conventional clinical imaging modalities could enable high-resolution, noninvasive quantification of RA in human tumors. This would be a major development for diagnostic imaging, permitting noninvasive molecular profiling of tumors and assessment of therapeutic-target engagement and providing substantially increased target-specific image contrast, even shortly after agent administration. However, with the exception of several novel approaches currently under development, most clinical modalities are incapable of routinely imaging multiple contrast agents simultaneously [14–21]. Furthermore, the availability of targeted/untargeted contrast agent pairs would be a challenge for most clinical imaging studies.

In this context, we propose a novel cross-modality approach to PAI which could accelerate the investigation of PAI using hybrid clinical modalities (such as PET/CT or PET/MRI). In this paradigm, the targeted and untargeted agents are imaged with separate modalities and analyzed using the traditional PAI quantitative framework to recover RA. To establish the feasibility of this approach, we acquired single-modality and cross-modality paired-agent data simultaneously in animal models bearing orthotopic tumors with an elevated expression of epidermal growth factor receptor (EGFR). This was accomplished using a hybrid MRI-fluorescence tomography instrument capable of imaging two spectrally distinct fluorescent agents (an EGFR-targeted agent and a non-targeted isotype) and a common gadolinium-based contrast agent (GBCA), which acts as a nonspecific extracellular contrast agent. The uptake kinetics of all three agents were imaged over 1 h post-administration and RA recovered by pairing the optical agents (single-modality PAI) or by pairing the targeted optical and GBCA (cross-modality PAI). A standard linear regression analysis comparing the recovered values of RA for the two methods was used to evaluate the feasibility of cross-modality PAI.

## Methods

### Experimental Design

This study compares receptor availability values recovered using an all-optical PAI approach and a cross-modality PAI approach from the same animals (*n* = 18). Each animal was co-administered a targeted optical contrast agent, an untargeted optical agent, and a GBCA. The kinetic behaviors of all three were imaged simultaneously for approximately 1 h using an MRI-coupled fluorescence tomography system [10]. The kinetics data is included as supplementary material (Table S1). This enabled RA to be determined using the two optical agents (single-modality PAI) and the targeted optical/GBCA (cross-modality PAI).

This study represents an extended analysis of an earlier study that evaluated single-modality PAI using the same animals [11]. As such, agent and animal preparation and fluorescence tomographic image reconstruction procedures are only briefly summarized here and referenced accordingly. Herein, we detail the recovery of cross-modality RA and the corresponding correlation analysis.

### Mouse Handling

Two cell lines were used in this study: U251 glioma cells (moderate EGFR expressing) (provided from Dr. Israel at Dartmouth College, Lebanon, NH) and 9L rat sarcoma cells (negative for EGFR expression) (provided from Dr. Wheeler at Wake Forest University, Winston-Salem, NC), providing a range of receptor expression profiles [8, 12, 22]. Cells were maintained in DMEM culture media supplemented with 5% fetal bovine serum and 1% antibiotic at 5% CO_2_, 37°C.

Animal studies were conducted in accordance with protocols approved by the Institutional Animal Care and Use Committee (IACUC) at Dartmouth College. Female nude mice were purchased from Charles River Laboratories (Wilmington, MD). Mice were orthotopically implanted with 1 × 10^6^ tumor cells, as previously described [23]. Animals were monitored for recovery post tumor implantation and continually monitored for neurological signs of tumor growth. Approximately 3–5 weeks post tumor implantation, mice were imaged with gadolinium-enhanced MRI (Gd-MRI) to confirm presence of orthotopic tumors.

### Imaging Agents

The targeted imaging agent was ABY-029, a GLP formulation of anti-EGFR affibody (Affibody AB (Solna, Sweden)) conjugated to IRDye 800CW Maleimide (LI-COR Biosciences, Inc., Lincoln, Nebraska) as described [24]. The untargeted counterpart was Affibody imaging agent, negative control bound to LI-COR’s IRDye 680RD [12]. For MRI-PAFT studies, the targeted and untargeted optical imaging agents (concentration of 0.2 nmol per agent) were mixed with an MRI contrast agent, Gadovist (0.5 mmol/kg) (Bayer, Leverkusen, Germany), in an injection solution of 200 μl per dose and administered to the animals via tail vein injection.

### Hybrid MRI-Fluorescence Tomography System

The MRI-coupled fluorescence tomography (FMT) system has been described in previous publications [10, 11, 25–28]. Briefly, the instrument consists of a clinical Philips Achieva 3.0 T scanner and a spectrometer-based FMT system that includes eight optical fibers extending into the bore of the MRI scanner and positioned around the head of the animal using a custom rodent optical/RF coil. Each fiber acts as a source or a detector, allowing an excitation source multiplexer to sequentially illuminate each fiber while the remaining seven fibers detect the transmitted light using high-sensitivity spectrometers. Optical filtering and spectral decomposition enable the separation of the two fluorescent agents prior to image reconstruction [27].

### Dynamic MRI-FMT Imaging of Three Agents:

After positioning the animal in the RF/optical coil, MRI and fluorescence tomography images were acquired once just prior to administration of the 3-contrast-agent cocktail (two fluorescent and one GBCA). Immediately after administration, dynamic data was acquired of all three agents concurrently for about 60 min. Dynamic GBCA data were acquired by repeating a T1-weighted turbo spin echo sequence (TR = 121 ms, TE = 10 ms, FOV = 90 mm × 90 mm, number of slices = 4, dimension of reconstruction matrix = 256 × 256, slice thickness = 1 mm, and flip angle = 90°, 89 s per frame). An additional high-resolution T1-weighted turbo spin echo sequence (TR = 744 ms, TE = 10 ms, FOV = 90 mm × 90 mm, number of slice = 30, dimension of reconstruction matrix = 256 × 256, slice thickness= 0.75 mm, and flip angle = 90°) was acquired for 15 min into the sequence to provide a template for the optical reconstruction. Optical data consisted of full tomographic projections of each spectral channel (i.e., for each optical imaging agent) acquired at about one frame per 108 s. Volumetric images of fluorescence intensities were reconstructed at each frame and for each agent using a hard-priors technique guided by an anatomically segmented high-resolution MRI volume [26]. This data processing technique produced the following three data sequences for the 1-h acquisition: (1) dynamic MRI scans of GBCA, (2) dynamic fluorescent intensity of the targeted fluorescent agent, and (3) dynamic fluorescent intensity of the untargeted fluorescent agent, in the tumor, brain tissue, and “outside of brain” tissue. Because acquisition points were not strictly simultaneous, data points along the sequence were linearly interpolated to align with each other prior to analysis. The kinetic curves of each agent were normalized to their own area under the curve (AUC) for comparison and correlation analysis.

### Recovery of Receptor Availability (RA) Using Paired-Agent Analysis

Paired-agent recovery of RA was accomplished using the snapshot technique [29], which is a simple approach that compares normalized targeted and untargeted signal intensities at a given time. The intensities for each agent at a given time, *t*, are normalized to the intensities from the first measurements: $\frac{T(t)}{T(0)}=\overset{\sim }{T}(t)$and $\frac{UT(t)}{UT(0)}=\overset{\sim }{UT}(t)$, where *T*(*t*) and *UT*(*t*) represent targeted and untargeted agents intensities at time *t* and $\overset{\sim }{T}(t)$ and $\overset{\sim }{UT}(t)$are normalized intensities for targeted and untargeted agents. *T*(0) and *UT*(0) are targeted and untargeted agent intensities at the first dynamic measurement after contrast administration. The snapshot RA, at time *t*, is calculated as follows:

1 $$ RA(t)=\frac{\overset{\sim }{T}(t)-\overset{\sim }{UT}(t)}{\overset{\sim }{UT}(t)} $$

The snapshot RA calculated using this equation is essentially identical to the “nondisplaceable binding potential” referred to in PET kinetic modeling, which is the product of the receptor concentration available to bind and the affinity of the imaging agent [30]. These values were computed for both the all-optical single-modality and optical/MRI cross-modality agent pairs. The snapshot approach provided estimates of RA recovered at each time point over the entire dynamic sequence.

### Correlation Analysis

To assess the correlation between single- and cross-modality PAI, a repeated measures correlation coefficient analysis was applied following the method reported by Bland and Altman for calculating correlation coefficient with repeated observations [31–33]. Specifically, analysis of covariance (ANCOVA) was performed to account for inter-individual variability among observations by providing parallel linear regression for individual samples. ANCOVA analyses were performed using the MATLAB (Matlab_R2020a) tool: *aoctool*. From the ANCOVA, regression sum of the squares (*SSR*) and error sum of squares (*SSE*) were calculated as:

2 $$ SSR={\sum}_{j=1}^n{\sum}_{i=1}^{m_j}{\left({\hat{y}}_{ij}-{\overline{y}}_j\right)}^2 $$

3 $$ SSE={\sum}_{j=1}^n{\sum}_{i=1}^{m_j}{\left({y}_{ij}-{\hat{y}}_{ij}\right)}^2 $$

for an experiment including *j* = *n* subjects with each subject provided *i* = *m*_*j*_ measurements. *SSR* is the sum of squared differences between the predicted value, ${\hat{y}}_{ij}$, and the mean of dependent variable for each participant, ${\overline{y}}_j$. *SSE* is the sum of squared differences between the dependent variables, *y*_*ij*_, and the regression-predicted values. The repeated measures correlation coefficient (*r*) was then calculated as a ratio of the regression sum of squares and the error sum of squares:

4 $$ r=\sqrt{\frac{SSR}{SSR+ SSE}} $$

The value of *r* does not change based on which variable is specified as dependent variable, since switching the dependent and independent variable in a regression model only changes the values of the sum of squares relatively. In this study, the cross-modality PAI results were used as the dependent variables, while the conventional PAI results were used as the independent variables. The significance, *p* value, is determined by the F test associated with the ANCOVA.

## Result

**Figure 1:**
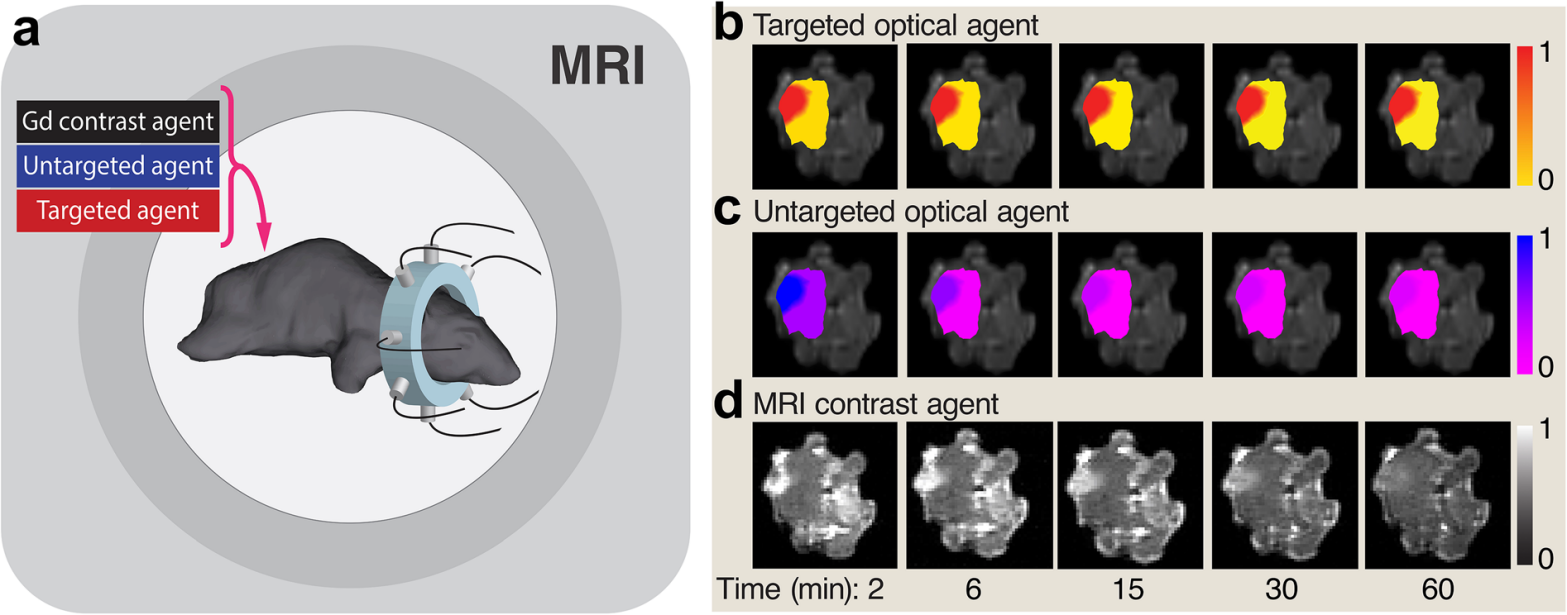
Simultaneous dynamic MRI and MRI-PAFT imaging. **a** Schematic diagram of MRI-PAFT animal interface inside the magnet bore showing optical fibers circumscribe the tumor site at different positions for tomographic reconstruction. Volumetric information of fluorescence intensities was acquired continuously at approximately 100 s per frame for approximately 60 min, producing dynamic image stacks overlaid with high-resolution MRI for targeted (**b**) and untargeted optical agents (**c**). Dynamic MRI utilizing a fast T1 MRI protocol is acquired simultaneously with fluorescence tomography to capture the GBCA signals (**d**).

An illustration of the MRI-PAFT system during data acquisition is shown in Figure 1a. Coronal cross sections of the fluorescence intensities inside the brain were overlaid on high-resolution MRI scans at selected frames over the sequence for visualization (Figure 1b, c). Corresponding slices of the dynamic MRI images taken at each selected frame are shown in Fig. 1d. This example indicates that both the untargeted fluorescent agent and the GBCA cleared steadily from the tumor over the hour, whereas the targeted agent signal remained relatively steady in the tumor over this interval.

**Figure 2:**
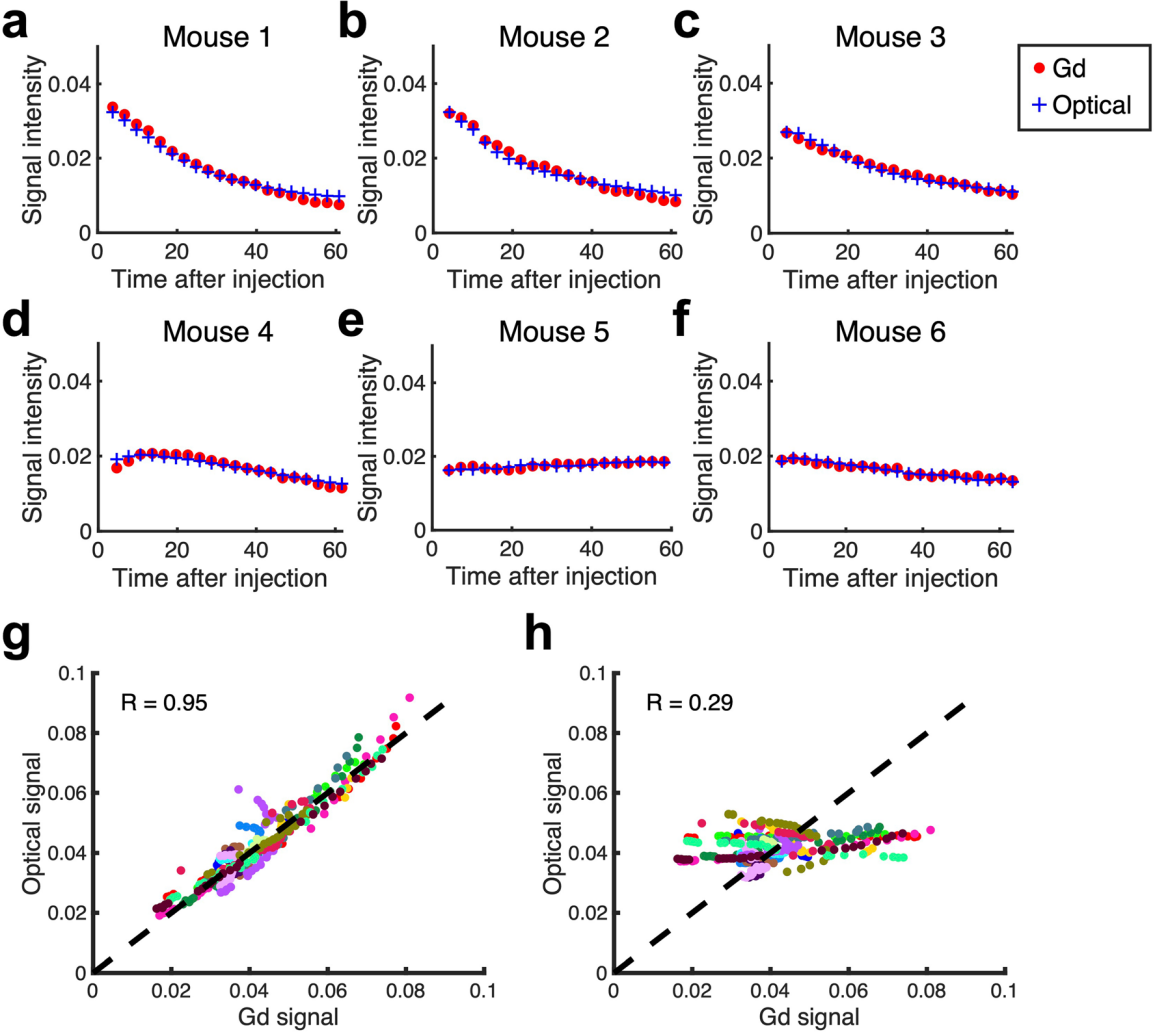
Uptake kinetics of GBCA and optical agents in tumors extracted from tomographic data. **a–f** Representative plots from 6 animals showed strong correspondence of normalized kinetics curves comparing GBCA agent and optical untargeted agent. **g** GBCA signal was plotted against optical untargeted agent’s signal at each time point showing a strong correlation (*r* = 0.95, *p* < 0.00001). **h** No significant correlation was observed from the scatter plot of GBCA signal and optical targeted agent’s signal (*r*= 0.29, *p* = 0.028). Each color represents data from different mice (n = 18).

Further evaluation is provided in Fig. 2a, b, c, d, e, f, which shows the mean signal intensity in the tumor volume over time for both the untargeted optical agent and GBCA in six representative animals. As observed, the kinetic curves show close correspondence between the optical untargeted agent and GBCA. Notably, this observation is consistent despite biological variations in contrast agent uptake between samples. Figure 2g provides a scatter plot of the untargeted optical imaging agent signal vs. the GBCA intensity for all time points of the samples. The repeated measures correlation coefficient was determined to be 0.95 (*p* < 0.00001), and the residual sum of squares (RSS) calculated between MRI readouts and fluorescence tomography signals was 0.0059. The EGFR-targeted optical agent curves were not correlated with the GBCA (*r* = 0.29, *p* = 0.028, and RSS = 0.054).

**Figure 3:**
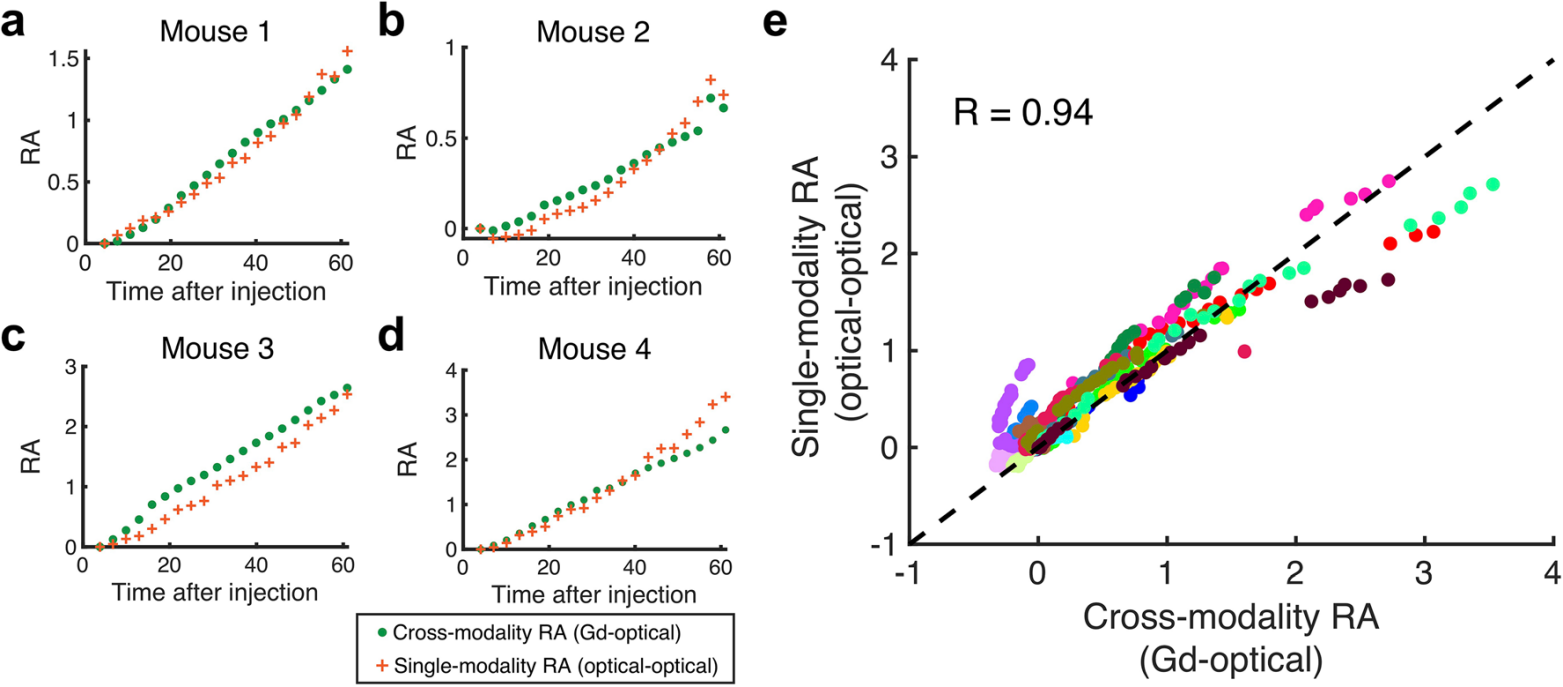
Estimating receptor availability using optical agents and GBCA. **a–d** Examples of snapshot receptor availability curves from 4 different animals showed that snapshot receptor availabilities were consistent with those computed using the single-modality (all-optical) technique. **e** Quantitative correlation analysis comparing receptor availabilities computed using the cross-modality and conventional single-modality data points (*r* =0.94, *p* < 0.00001). Each color represents data from different mice (n = 18).

Finally, we compared values of receptor availability recovered using the conventional approach and the cross-modality implementation. The former paired the targeted fluorescent agent with the untargeted fluorescent agent, while the latter used the same targeted fluorescent data, but substituted the untargeted agent with the GBCA curves. Receptor availability was computed using the snapshot approach, which provided RA at each time point in the kinetic sequence. Representative plots of conventional and cross-modality RA values are shown in Fig. 3a, b, c, d. As observed, values of RA recovered using the cross-modality approach tracked closely with the conventional approach. A correlation plot between the two techniques for all animals and all time points is shown in Fig. 3e and confirms this trend. The repeated measures correlation coefficient between conventional and cross-modality RA was found to be *r* = 0.94 (*p* < 0.00001), and root mean square error (RMSE) between the two techniques was 0.25 ± 0.14 (standard deviation).

## Discussion

Paired-agent imaging using two fluorescent agents has emerged as an effective strategy for rapidly quantifying the availability of receptor targets in solid tumors. However, the use of optical modalities limits the application of this strategy to superficial tissues, invasive techniques, or shallow subsurface structures that can be measured using various tomographic techniques. Towards expanding the application of paired agent techniques into more conventional modalities, this study examined the feasibility of deploying the approach in a cross-modality imaging scheme combining clinical MRI contrast agent with optical imaging agents to quantify glioma tumor receptor availability, noninvasively. The central finding of the study was that receptor availability values recovered using the cross-modality approach were highly correlated to the conventional, single-modality approach, suggesting that cross-modality PAI is feasible and potentially translatable to existing imaging modalities.

Although the high correlation between single- and cross-modality approaches reported in this study is encouraging for further development of the strategy, we observed that the agreement between the two techniques started to diverge for some subjects at later time points/higher RA values (represented by red, brown, and green dots in Fig. 3e). Tumor volumes for these animals were all well within one standard deviation of the mean tumor volume of all subjects, indicating this behavior is likely not a feature of tumor size. Rather, the behavior seems to be associated with higher RA values. Further investigation in a larger group of animals may help explain this behavior and identify time intervals over which the two techniques agree.

An important advantage of paired-agent imaging is that it provides quantitative receptor concentration imaging shortly after contrast agent administration, when signal tends to be high. This is generally intractable for single-agent imaging in which contrast only emerges at much later time points, when agent has had a chance to clear from normal tissues. Even at these long time points, a significant proportion of the retained agent in the tumor can be due to nonspecific retention and thus is generally not considered quantitative. By contrast, paired-agent techniques can produce high-contrast, quantitative images of specific binding within minutes of administration [11]. This feature could be particularly impactful for radionuclides which lose signal strength through decay.

In this study, fluorescence tomographic imaging was chosen as one of the modalities in the cross-modality paradigm. Although the limitations on volumetric imaging discussed above apply to these data, this strategy also offers a unique opportunity to validate the cross-modality approach. Specifically, this approach allowed the direct comparison of cross-modality and single-modality PAI recovered from data acquired simultaneously in the same animals. This is a critical validating step in the further development of cross-modality PAI. The central limitation of this feasibility study is that it did not address potential differences in the non-specific kinetics of the agents. If the untargeted agent exhibits different hemodynamics, vascular permeability, and level of non-specific uptake as the targeted agent, the paired-agent scheme would not accurately estimate receptor expression. The observed similarities between the untargeted optical agent and GBCA were largely responsible for the strong correlation between single- and cross-modality RA values. However, several previous reports indicate that deconvolution and/or normalization strategies using appropriately selected normal tissue volumes can compensate for differences in kinetics between the targeted and control agent pair, effectively relaxing the constraints on agent selection. Validating these compensatory strategies in the cross-modality framework will be an important goal for future studies.

Another area of investigation for any paired-agent approach, whether deployed in a single- or cross-modality framework, will be the determination of the minimum detectable change in RA. We have previously shown that the single-modality approach can rapidly detect large changes in RA[11]; however, the capacity to distinguish small, potentially consequential differences in RA has not been fully explored. For any implementation of PAI, this metric will be contingent on the specific modalities used. Spatial resolution, sensitivity to the imaging agent, contribution of background signal, and behavior of the contrast agents will all impact accuracy of the recovered RA values. Evaluating RA sensitivity against quantitative receptor expression assays of *ex vivo* specimens will be a challenging yet important step to establish the sensitivity metric.

Other novel approaches for clinical receptor imaging are under development. Among these, immuno-PET is the most advanced. These studies often seek to report on receptor occupancy by utilizing pre- and post-drug administration competitive binding metrics [34, 35]. Unlike paired-agent techniques, which require only one imaging session, this pre- and post- comparison is central for single-agent immuno-PET. The emerging chemical exchange saturation transfer (CEST) techniques also enable imaging multiple MRI contrast agents, representing another potential avenue for applying the paired-agent framework, provided sensitivity challenges can be overcome [19, 20]. Studies using dual-isotope PET/SPECT demonstrate a potential path towards quantifying RA using a cross-modality PAI framework [21, 36–38].

The results presented herein are the first to establish the feasibility of cross-modality paired-agent imaging and thus represent an important development that could facilitate rapid, noninvasive quantification of drug targets using conventional imaging modalities, such as PET/MRI and PET/CT, and existing contrast agents. This capability could enable stratification of patients for treatment and monitoring of receptor occupancy during treatment and could facilitate the study of receptor behavior and drug engagement in preclinical research.

## Supplementary information

ESM 1 (DOCX 51 kb)
