# Supplementary material for: Examining the Feasibility of Quantifying Receptor Availability Using Cross-Modality Paired-Agent Imaging
Source: Mol Imaging Biol. 2021 Jul 20;24(1):23–30. doi: 10.1007/s11307-021-01629-6 (PMC8760219; doi:10.1007/s11307-021-01629-6)
Supplement: Supplementary file 1 — (DOCX 51 kb) [file 11307_2021_1629_MOESM1_ESM.docx]

Electronic Supplementary Material

**Title:** Examining the feasibility of quantifying receptor availability using cross-modality paired agent imaging

**Journal:** Molecular Imaging and Biology

**Authors:** Boyu Meng^a^, Negar Sadeghipour^b^, Margaret R. Folaron^a^, Rendall R. Strawbridge^a^, Kimberley S. Samkoe^a,c^, Kenneth M. Tichauer^b^, Scott C. Davis^a^

^a^Thayer School of Engineering, Dartmouth College, Hanover, NH 03755; ^b^Biomedical Engineering, Illinois Institute of Technology, Chicago, IL 60616; ^c^Geisel School of Medicine, Dartmouth College, Hanover, NH 03755;

**Running title:** Dynamic MRI and fluorescence tomography hybrid imaging

**Category:** Brief article

**Corresponding author:**

Scott C. Davis, PhD

Thayer School of Engineering, Dartmouth College, Hanover, NH 03755

Phone: (603) 646-9684 Fax: (603) 646-3856

Email: scott.c.davis@dartmouth.edu

**Dataset:**

**Table S1: Kinetics dataset used in this study for 18 mice including acquisition time in minutes, signal intensities in tumors for optical targeted agent, optical untargeted agent, and GBCA.**

| Mouse1 | data |  |  |
| --- | --- | --- | --- |
| Acq_time (min) | Optical Targeted | Optical untargeted | GBCA |
| 3.97E+00 | 0.00004054 | 0.00002936 | 272.029506 |
| 5.53E+00 | 0.00004075 | 0.00002894 | 277.612655 |
| 7.08E+00 | 0.00003973 | 0.00002848 | 279.353834 |
| 8.58E+00 | 0.00003929 | 0.00002852 | 278.570471 |
| 1.11E+01 | 0.0000389 | 0.0000289 | 278.187463 |
| 1.26E+01 | 0.0000384 | 0.00002892 | 276.837374 |
| 1.42E+01 | 0.00003814 | 0.00002868 | 275.162034 |
| 1.58E+01 | 0.0000373 | 0.00002881 | 270.793372 |
| 2.61E+01 | 0.00003473 | 0.0000301 | 251.736842 |
| 2.87E+01 | 0.0000342 | 0.00003018 | 249.687167 |
| 3.03E+01 | 0.0000339 | 0.00003021 | 243.837769 |
| 3.19E+01 | 0.00003356 | 0.00003031 | 241.468165 |
| 3.36E+01 | 0.00003322 | 0.00003047 | 236.800439 |
| 3.51E+01 | 0.00003288 | 0.00003063 | 232.430317 |
| 3.76E+01 | 0.00003181 | 0.00003085 | 226.302977 |
| 3.91E+01 | 0.00003192 | 0.00003113 | 219.167948 |
| 4.08E+01 | 0.00003163 | 0.00003144 | 217.717918 |
| 4.24E+01 | 0.00003116 | 0.00003163 | 213.780603 |
| 4.41E+01 | 0.0000308 | 0.00003183 | 211.815789 |
| 5.56E+01 | 0.00003012 | 0.00003359 | 181.330968 |
| 5.72E+01 | 0.00002922 | 0.00003432 | 178.733254 |
| 5.87E+01 | 0.00002736 | 0.00003402 | 178.397661 |
| 6.04E+01 | 0.00002704 | 0.00003237 | 174.647129 |
| 6.20E+01 | 0.00002683 | 0.00003283 | 171.668244 |
| Mouse2 | data |  |  |
| Acq_time (min) | Optical Targeted | Optical untargeted | GBCA |
| 4.35E+00 | 0.00003252 | 0.00002894 | 193.642857 |
| 5.95E+00 | 0.00003091 | 0.0000288 | 191.973716 |
| 7.55E+00 | 0.00002955 | 0.00002897 | 187.809992 |
| 9.17E+00 | 0.00002827 | 0.00002915 | 181.161517 |
| 1.18E+01 | 0.00002562 | 0.00002899 | 171.163122 |
| 1.35E+01 | 0.00002424 | 0.00002892 | 163.380682 |
| 1.52E+01 | 0.00002316 | 0.00002905 | 157.396669 |
| 2.61E+01 | 0.00001704 | 0.00002907 | 120.952648 |
| 2.78E+01 | 0.00001644 | 0.00002904 | 115.564369 |
| 3.04E+01 | 0.00001572 | 0.00002912 | 108.947674 |
| 3.21E+01 | 0.00001511 | 0.00002911 | 104.225291 |
| 3.37E+01 | 0.00001467 | 0.00002908 | 99.1287375 |
| 3.54E+01 | 0.00001426 | 0.00002894 | 92.6108804 |
| 3.71E+01 | 0.00001406 | 0.00002878 | 87.3550664 |
| 3.97E+01 | 0.00001328 | 0.00002861 | 82.3741597 |
| 4.13E+01 | 0.00001292 | 0.00002868 | 79.6100498 |
| 4.30E+01 | 0.00001258 | 0.00002877 | 74.6976744 |
| 4.47E+01 | 0.00001231 | 0.00002882 | 71.603613 |
| 4.63E+01 | 0.00001207 | 0.00002889 | 69.2304817 |
| 5.82E+01 | 0.00001038 | 0.00002866 | 51.3745847 |
| 5.98E+01 | 0.0000101 | 0.00002867 | 48.8085548 |
| 6.15E+01 | 0.00001001 | 0.00002873 | 47.2531146 |
| Mouse3 | data |  |  |
| Acq_time (min) | Optical Targeted | Optical untargeted | GBCA |
| 4.47E+00 | 0.00004332 | 0.00003465 | 208.787679 |
| 6.12E+00 | 0.00004375 | 0.00003475 | 201.867555 |
| 7.78E+00 | 0.00004256 | 0.00003487 | 195.457187 |
| 9.43E+00 | 0.00004092 | 0.00003465 | 186.719489 |
| 1.22E+01 | 0.00003868 | 0.00003416 | 179.029833 |
| 1.38E+01 | 0.00003753 | 0.00003412 | 172.61914 |
| 1.56E+01 | 0.00003622 | 0.00003405 | 170.059387 |
| 2.65E+01 | 0.00002797 | 0.00003341 | 141.535451 |
| 2.81E+01 | 0.00002705 | 0.00003349 | 136.800077 |
| 3.08E+01 | 0.0000259 | 0.00003346 | 132.425417 |
| 3.24E+01 | 0.00002494 | 0.00003352 | 128.709802 |
| 3.41E+01 | 0.00002441 | 0.0000335 | 123.501744 |
| 3.57E+01 | 0.00002357 | 0.0000337 | 121.567006 |
| 3.74E+01 | 0.00002323 | 0.00003383 | 120.399458 |
| 4.00E+01 | 0.00002246 | 0.00003398 | 113.371561 |
| 4.17E+01 | 0.00002206 | 0.00003408 | 112.456025 |
| 4.33E+01 | 0.00002156 | 0.00003405 | 109.810539 |
| 4.50E+01 | 0.0000217 | 0.00003396 | 104.640837 |
| 4.67E+01 | 0.00002123 | 0.00003413 | 103.490508 |
| 5.86E+01 | 0.00001832 | 0.00003424 | 87.1693142 |
| 6.03E+01 | 0.00001795 | 0.00003424 | 82.6888715 |
| 6.20E+01 | 0.00001765 | 0.00003418 | 80.4366525 |
| Mouse4 | data |  |  |
| Acq_time (min) | Optical Targeted | Optical untargeted | GBCA |
| 3.92E+00 | 0.00007054 | 0.00006622 | 213.274113 |
| 5.32E+00 | 0.00006552 | 0.0000644 | 202.522609 |
| 6.73E+00 | 0.00005978 | 0.00006278 | 193.576322 |
| 8.23E+00 | 0.00005543 | 0.00006147 | 183.999452 |
| 1.08E+01 | 0.00004903 | 0.00006073 | 169.84023 |
| 1.24E+01 | 0.00004422 | 0.00005926 | 162.365854 |
| 1.39E+01 | 0.00004073 | 0.00005806 | 155.760255 |
| 1.54E+01 | 0.000037 | 0.00005783 | 146.764867 |
| 2.57E+01 | 0.00002741 | 0.0000547 | 104.729197 |
| 2.82E+01 | 0.00002615 | 0.00005416 | 97.1277062 |
| 2.97E+01 | 0.00002515 | 0.00005402 | 89.9512195 |
| 3.13E+01 | 0.00002452 | 0.00005385 | 85.3968211 |
| 3.28E+01 | 0.00002369 | 0.00005366 | 84.097835 |
| 3.44E+01 | 0.00002283 | 0.0000533 | 80.7259523 |
| 3.71E+01 | 0.00002174 | 0.00005309 | 77.5083149 |
| 3.86E+01 | 0.00002128 | 0.00005301 | 74.2625377 |
| 4.01E+01 | 0.00002054 | 0.00005283 | 72.4439573 |
| 4.16E+01 | 0.00001968 | 0.00005258 | 69.718553 |
| 4.31E+01 | 0.00001963 | 0.00005238 | 69.9569745 |
| 5.43E+01 | 0.00001634 | 0.00005216 | 54.5180155 |
| 5.58E+01 | 0.00001597 | 0.00005187 | 53.1986846 |
| 5.73E+01 | 0.00001579 | 0.00005176 | 52.7196492 |
| 5.88E+01 | 0.00001546 | 0.00005176 | 48.6544259 |
| 6.03E+01 | 0.00001525 | 0.00005169 | 47.0641272 |
| 6.29E+01 | 0.00001469 | 0.00005168 | 44.7284188 |
| Mouse5 | data |  |  |
| Acq_time (min) | Optical Targeted | Optical untargeted | GBCA |
| 4.38E+00 | 0.00001438 | 0.00001418 | 93.8392857 |
| 6.12E+00 | 0.00001362 | 0.00001387 | 92.2857143 |
| 7.98E+00 | 0.00001293 | 0.00001372 | 91.4692691 |
| 9.68E+00 | 0.00001242 | 0.0000137 | 71.0214286 |
| 1.24E+01 | 0.0000114 | 0.00001377 | 67.89701 |
| 1.41E+01 | 0.00001109 | 0.00001383 | 68.4731451 |
| 1.59E+01 | 0.0000108 | 0.00001394 | 68.3341977 |
| 2.53E+01 | 0.00000979 | 0.0000149 | 65.0131506 |
| 2.71E+01 | 0.00000973 | 0.00001513 | 64.3887043 |
| 2.88E+01 | 0.00000962 | 0.00001535 | 63.4791317 |
| 3.14E+01 | 0.00000949 | 0.00001562 | 61.469546 |
| 3.31E+01 | 0.00000945 | 0.00001585 | 60.8089701 |
| 3.48E+01 | 0.00000928 | 0.0000161 | 60.0863787 |
| 3.65E+01 | 0.00000918 | 0.00001629 | 57.8468992 |
| 3.82E+01 | 0.00000907 | 0.00001644 | 57.8504983 |
| 4.08E+01 | 0.00000901 | 0.00001667 | 58.0014006 |
| 4.25E+01 | 0.00000885 | 0.00001678 | 56.6459026 |
| 4.42E+01 | 0.00000878 | 0.00001686 | 56.5728129 |
| 4.59E+01 | 0.00000866 | 0.00001695 | 56.2439092 |
| 4.77E+01 | 0.00000888 | 0.00001699 | 55.7087486 |
| 5.96E+01 | 0.00000748 | 0.00001723 | 46.2356036 |
| 6.14E+01 | 0.00000735 | 0.00001722 | 46.47799 |
| Mouse6 | data |  |  |
| Acq_time (min) | Optical Targeted | Optical untargeted | GBCA |
| 3.75E+00 | 0.00004939 | 0.00003164 | 137.764706 |
| 5.20E+00 | 0.00004895 | 0.00002956 | 143.898215 |
| 6.67E+00 | 0.00004868 | 0.00002898 | 150.841154 |
| 8.12E+00 | 0.00004842 | 0.00002862 | 152.799515 |
| 1.06E+01 | 0.00004769 | 0.00002806 | 156.14082 |
| 1.21E+01 | 0.00004718 | 0.00002791 | 157.074906 |
| 1.36E+01 | 0.00004649 | 0.00002772 | 156.068077 |
| 1.51E+01 | 0.00004594 | 0.00002745 | 156.260189 |
| 2.50E+01 | 0.00004156 | 0.00002695 | 155.385941 |
| 2.75E+01 | 0.00004061 | 0.00002681 | 153.171403 |
| 2.90E+01 | 0.00003985 | 0.00002673 | 151.011016 |
| 3.05E+01 | 0.00003917 | 0.0000267 | 149.742014 |
| 3.20E+01 | 0.00003838 | 0.00002668 | 146.295009 |
| 3.35E+01 | 0.00003765 | 0.00002667 | 145.118519 |
| 3.59E+01 | 0.00003658 | 0.0000266 | 144.019608 |
| 3.74E+01 | 0.00003599 | 0.00002651 | 141.072263 |
| 3.89E+01 | 0.00003536 | 0.00002643 | 139.790042 |
| 4.04E+01 | 0.00003495 | 0.00002632 | 141.023794 |
| 4.20E+01 | 0.00003455 | 0.00002628 | 137.791143 |
| 5.32E+01 | 0.00003088 | 0.00002596 | 127.209297 |
| 5.47E+01 | 0.0000305 | 0.00002583 | 125.207535 |
| 5.63E+01 | 0.00003012 | 0.00002579 | 123.733862 |
| 5.79E+01 | 0.0000296 | 0.00002581 | 122.990306 |
| 5.95E+01 | 0.00002929 | 0.00002583 | 121.666226 |
| 6.19E+01 | 0.00002858 | 0.00002588 | 120.072924 |
| 6.35E+01 | 0.00002831 | 0.00002581 | 118.495263 |
| Mouse7 | data |  |  |
| Acq_time (min) | Optical Targeted | Optical untargeted | GBCA |
| 4.08E+00 | 0.00005192 | 0.00003416 | 188.273336 |
| 5.65E+00 | 0.0000496 | 0.00003343 | 187.14758 |
| 7.20E+00 | 0.00004657 | 0.00003276 | 181.946197 |
| 8.75E+00 | 0.00004331 | 0.00003214 | 176.495678 |
| 1.12E+01 | 0.00003997 | 0.00003139 | 168.079516 |
| 1.27E+01 | 0.00003821 | 0.00003104 | 161.408816 |
| 1.43E+01 | 0.00003577 | 0.00003076 | 157.871435 |
| 1.59E+01 | 0.00003417 | 0.00003054 | 145.001885 |
| 2.63E+01 | 0.00002589 | 0.00002862 | 110.50497 |
| 2.88E+01 | 0.00002458 | 0.00002841 | 102.423077 |
| 3.04E+01 | 0.00002384 | 0.00002829 | 101.428263 |
| 3.20E+01 | 0.00002315 | 0.00002825 | 99.5583405 |
| 3.36E+01 | 0.00002246 | 0.0000282 | 99.2973207 |
| 3.52E+01 | 0.00002189 | 0.00002809 | 97.0421348 |
| 3.78E+01 | 0.000021 | 0.00002797 | 95.18172 |
| 3.94E+01 | 0.00002051 | 0.0000279 | 93.3057476 |
| 41 | 0.00002003 | 0.00002781 | 92.0905359 |
| 4.26E+01 | 0.00001961 | 0.00002773 | 89.9727191 |
| 4.42E+01 | 0.00001915 | 0.00002762 | 87.1426655 |
| 5.59E+01 | 0.0000165 | 0.00002723 | 71.2285068 |
| 5.75E+01 | 0.00001625 | 0.00002724 | 70.0149821 |
| 5.91E+01 | 0.00001595 | 0.00002724 | 65.5769231 |
| 6.08E+01 | 0.00001558 | 0.00002734 | 68.1395349 |
| 6.24E+01 | 0.00001511 | 0.00002737 | 63.7602862 |
| Mouse8 | data |  |  |
| Acq_time (min) | Optical Targeted | Optical untargeted | GBCA |
| 4.13E+00 | 0.00008836 | 0.00007063 | 28.1723044 |
| 5.53E+00 | 0.00008844 | 0.00007034 | 29.3646934 |
| 7.10E+00 | 0.00008809 | 0.00006973 | 29.5491543 |
| 8.52E+00 | 0.00008847 | 0.0000692 | 28.461945 |
| 1.10E+01 | 0.00008697 | 0.00006953 | 26.5560254 |
| 1.24E+01 | 0.00008653 | 0.00006989 | 28.3763214 |
| 1.38E+01 | 0.00008627 | 0.00006984 | 28.5665962 |
| 1.52E+01 | 0.000085 | 0.00006983 | 29.329808 |
| 2.52E+01 | 0.00008011 | 0.00006889 | 30.7249475 |
| 2.78E+01 | 0.00007963 | 0.00006854 | 27.8562368 |
| 2.93E+01 | 0.00007853 | 0.00006816 | 30.0363636 |
| 3.08E+01 | 0.00007757 | 0.00006866 | 30.5454545 |
| 3.39E+01 | 0.00007683 | 0.00006929 | 28.1104651 |
| 3.65E+01 | 0.0000759 | 0.00006883 | 29.1960888 |
| 3.80E+01 | 0.00007538 | 0.00006876 | 30.6310782 |
| 3.95E+01 | 0.00007478 | 0.00006878 | 29.0048128 |
| 4.10E+01 | 0.00007383 | 0.00006876 | 30.2114165 |
| 4.35E+01 | 0.00007358 | 0.00006856 | 29.552094 |
| 4.50E+01 | 0.00007326 | 0.00006832 | 32.0190275 |
| 4.65E+01 | 0.00007253 | 0.00006884 | 29.6596195 |
| 4.80E+01 | 0.00007251 | 0.00006899 | 30.2240642 |
| 4.95E+01 | 0.00007188 | 0.00006877 | 30.3208245 |
| 5.21E+01 | 0.00007131 | 0.00006902 | 28.1940756 |
| 5.36E+01 | 0.00006996 | 0.00006902 | 26.6343434 |
| 5.51E+01 | 0.00006927 | 0.00006881 | 27.5278075 |
| 5.66E+01 | 0.00006856 | 0.00006888 | 26.9926004 |
| 5.81E+01 | 0.00006824 | 0.00006888 | 29.4714588 |
| Mouse9 | data |  |  |
| Acq_time (min) | Optical Targeted | Optical untargeted | GBCA |
| 3.62E+00 | 0.00007244 | 0.00005933 | 104.448837 |
| 4.97E+00 | 0.00007048 | 0.00005758 | 102.59402 |
| 6.33E+00 | 0.00007017 | 0.00005635 | 100.162791 |
| 7.68E+00 | 0.00006962 | 0.00005575 | 103.557311 |
| 9.90E+00 | 0.00006894 | 0.00005479 | 101.597342 |
| 1.14E+01 | 0.00006727 | 0.00005415 | 101.540199 |
| 1.28E+01 | 0.00006739 | 0.00005361 | 101.927243 |
| 1.42E+01 | 0.00006699 | 0.00005264 | 102.039867 |
| 1.56E+01 | 0.00006671 | 0.00005189 | 103.065064 |
| 2.56E+01 | 0.00006602 | 0.0000495 | 108.322689 |
| 2.70E+01 | 0.00006373 | 0.00004909 | 112.405316 |
| 2.83E+01 | 0.00006381 | 0.00004906 | 109.21794 |
| 3.09E+01 | 0.0000628 | 0.00004893 | 107.499003 |
| 3.31E+01 | 0.0000619 | 0.00004774 | 107.128571 |
| 3.44E+01 | 0.0000619 | 0.00004718 | 109.712292 |
| 3.58E+01 | 0.00006107 | 0.0000463 | 111.102857 |
| 3.72E+01 | 0.00006117 | 0.0000458 | 109.506977 |
| 3.94E+01 | 0.00006062 | 0.00004578 | 107.962791 |
| 4.07E+01 | 0.00006069 | 0.00004565 | 109.924252 |
| 4.21E+01 | 0.00006034 | 0.00004535 | 110.162126 |
| 4.35E+01 | 0.00005994 | 0.00004514 | 110.007973 |
| 4.48E+01 | 0.00005953 | 0.00004509 | 111.121681 |
| 4.70E+01 | 0.00005925 | 0.00004483 | 106.971429 |
| 4.84E+01 | 0.00005901 | 0.00004446 | 105.853156 |
| 4.98E+01 | 0.00005844 | 0.00004422 | 107.81495 |
| 5.12E+01 | 0.00005787 | 0.00004389 | 102.033223 |
| 5.26E+01 | 0.00005747 | 0.0000438 | 103.040336 |
| 5.49E+01 | 0.00005696 | 0.00004364 | 102.623256 |
| Mouse10 | data |  |  |
| Acq_time (min) | Optical Targeted | Optical untargeted | GBCA |
| 4.43E+00 | 0.00007049 | 0.00004583 | 93.177167 |
| 6.10E+00 | 0.00007037 | 0.00004504 | 93.8053476 |
| 7.75E+00 | 0.00007062 | 0.00004453 | 97.2701903 |
| 9.40E+00 | 0.00007051 | 0.00004452 | 96.3828753 |
| 1.21E+01 | 0.00006886 | 0.00004472 | 98.7803383 |
| 1.37E+01 | 0.00006904 | 0.00004496 | 100.021564 |
| 1.54E+01 | 0.00006881 | 0.00004514 | 96.3146715 |
| 2.48E+01 | 0.00006613 | 0.00004516 | 94.690189 |
| 2.64E+01 | 0.0000651 | 0.00004494 | 95.2752643 |
| 2.81E+01 | 0.00006411 | 0.00004484 | 95.3549683 |
| 3.08E+01 | 0.00006352 | 0.00004455 | 98.4395722 |
| 3.24E+01 | 0.00006336 | 0.00004457 | 94.0325581 |
| 3.41E+01 | 0.00006368 | 0.00004473 | 93.3610994 |
| 3.71E+01 | 0.0000655 | 0.00004494 | 94.6226216 |
| 3.98E+01 | 0.00006502 | 0.00004487 | 97.7420719 |
| 4.14E+01 | 0.0000645 | 0.00004473 | 96.7585624 |
| 4.31E+01 | 0.00006434 | 0.00004473 | 94.7197861 |
| 4.48E+01 | 0.00006393 | 0.00004473 | 95.4735729 |
| 4.74E+01 | 0.00006325 | 0.00004469 | 94.8676533 |
| 4.91E+01 | 0.00006278 | 0.00004471 | 82.3885835 |
| 5.07E+01 | 0.00006247 | 0.00004475 | 80.8790603 |
| 5.24E+01 | 0.00006221 | 0.00004468 | 74.3556025 |
| 5.40E+01 | 0.00006206 | 0.00004475 | 78.5306554 |
| 5.67E+01 | 0.0000614 | 0.00004505 | 75.2733615 |
| 5.85E+01 | 0.0000612 | 0.00004484 | 82.3382664 |
| Mouse11 | data |  |  |
| Acq_time (min) | Optical Targeted | Optical untargeted | GBCA |
| 4.50E+00 | 0.00001936 | 0.00001885 | 102.725 |
| 6.15E+00 | 0.00001832 | 0.00001853 | 99.1016854 |
| 7.92E+00 | 0.00001736 | 0.00001821 | 98.1758427 |
| 9.57E+00 | 0.00001646 | 0.00001798 | 91.1134832 |
| 1.24E+01 | 0.00001544 | 0.00001801 | 87.9477528 |
| 1.40E+01 | 0.00001486 | 0.00001807 | 80.805618 |
| 1.57E+01 | 0.00001451 | 0.00001793 | 78.1428105 |
| 2.51E+01 | 0.00001206 | 0.00001753 | 77.1272876 |
| 2.69E+01 | 0.00001174 | 0.00001739 | 74.6429412 |
| 2.85E+01 | 0.00001147 | 0.00001737 | 74.9587209 |
| 3.12E+01 | 0.00001101 | 0.00001745 | 71.3616279 |
| 3.29E+01 | 0.00001077 | 0.00001745 | 70.4773256 |
| 3.45E+01 | 0.00001057 | 0.00001746 | 66.7788235 |
| 3.62E+01 | 0.00001034 | 0.00001744 | 65.9156977 |
| 3.78E+01 | 0.00001011 | 0.00001736 | 61.0494186 |
| 4.05E+01 | 0.00000979 | 0.00001726 | 61.9593023 |
| 4.22E+01 | 0.00000962 | 0.0000172 | 61.2423529 |
| 4.38E+01 | 0.00000945 | 0.00001721 | 58.3860465 |
| 4.55E+01 | 0.0000093 | 0.00001724 | 56.8215116 |
| 4.72E+01 | 0.00000909 | 0.00001721 | 55.552907 |
| 5.93E+01 | 0.00000815 | 0.00001706 | 45.6313954 |
| 6.09E+01 | 0.000008 | 0.00001701 | 44.5976744 |
| Mouse12 | data |  |  |
| Acq_time (min) | Optical Targeted | Optical untargeted | GBCA |
| 3.87E+00 | 0.00004953 | 0.0000416 | 72.9654423 |
| 5.43E+00 | 0.00004954 | 0.00004009 | 75.0936753 |
| 7 | 0.00004986 | 0.00003914 | 75.6978918 |
| 8.55E+00 | 0.00004984 | 0.00003845 | 78.3935735 |
| 1.11E+01 | 0.00004999 | 0.00003771 | 80.7271868 |
| 1.26E+01 | 0.00005015 | 0.00003715 | 85.6593033 |
| 1.43E+01 | 0.00005015 | 0.00003652 | 81.8085687 |
| 1.58E+01 | 0.00004975 | 0.00003607 | 78.6347212 |
| 2.62E+01 | 0.00004921 | 0.00003342 | 81.8413339 |
| 2.89E+01 | 0.00004886 | 0.00003345 | 84.764255 |
| 3.05E+01 | 0.00004885 | 0.00003337 | 87.3361336 |
| 3.20E+01 | 0.00004838 | 0.00003328 | 84.8393363 |
| 3.49E+01 | 0.00004764 | 0.00003329 | 87.2413105 |
| 3.75E+01 | 0.00004668 | 0.00003285 | 85.7002974 |
| 3.90E+01 | 0.00004619 | 0.00003257 | 83.3301456 |
| 4.06E+01 | 0.0000454 | 0.00003244 | 83.3042109 |
| 4.21E+01 | 0.00004467 | 0.00003242 | 80.0667857 |
| 4.47E+01 | 0.00004369 | 0.00003224 | 78.6760475 |
| 4.64E+01 | 0.00004326 | 0.00003199 | 79.8509923 |
| 4.80E+01 | 0.00004225 | 0.00003138 | 79.4443978 |
| 4.96E+01 | 0.00004188 | 0.00003136 | 78.9685642 |
| 5.13E+01 | 0.00004112 | 0.00003137 | 78.9106374 |
| 5.39E+01 | 0.00004016 | 0.00003084 | 78.6476853 |
| 5.55E+01 | 0.00003963 | 0.00003129 | 78.4964138 |
| 5.71E+01 | 0.00003921 | 0.00003072 | 77.7555392 |
| 5.87E+01 | 0.00003845 | 0.00003012 | 75.7499725 |
| 6.03E+01 | 0.00003779 | 0.00003037 | 74.1643121 |
| 6.29E+01 | 0.00003658 | 0.00003094 | 72.8849102 |
| Mouse13 | data |  |  |
| Acq_time (min) | Optical Targeted | Optical untargeted | GBCA |
| 9.15E+00 | 0.000055 | 0.00004726 | 106.264045 |
| 1.07E+01 | 0.00005397 | 0.00004676 | 108.343669 |
| 1.22E+01 | 0.00005583 | 0.00004637 | 111.626615 |
| 1.38E+01 | 0.00005508 | 0.00004585 | 112.966667 |
| 1.63E+01 | 0.00005621 | 0.00004487 | 113.613695 |
| 1.78E+01 | 0.00005706 | 0.00004443 | 109.55168 |
| 1.93E+01 | 0.0000573 | 0.00004421 | 111.321705 |
| 2.08E+01 | 0.00005584 | 0.00004404 | 110.816465 |
| 3.10E+01 | 0.00005981 | 0.00004394 | 115.306202 |
| 3.35E+01 | 0.00005979 | 0.00004385 | 114.250646 |
| 3.50E+01 | 0.00005781 | 0.00004362 | 114.959948 |
| 3.65E+01 | 0.00005896 | 0.00004349 | 117.376615 |
| 3.80E+01 | 0.00005835 | 0.00004353 | 115.895995 |
| 3.95E+01 | 0.00005911 | 0.0000435 | 117.54183 |
| 4.20E+01 | 0.00005908 | 0.0000436 | 117.846899 |
| 4.35E+01 | 0.00005967 | 0.00004367 | 119.9677 |
| 4.50E+01 | 0.0000599 | 0.00004365 | 119.223514 |
| 4.65E+01 | 0.00006116 | 0.00004354 | 120.071059 |
| 48 | 0.00006167 | 0.00004349 | 119.707843 |
| 5.90E+01 | 0.00006311 | 0.00004377 | 116.381137 |
| 6.05E+01 | 0.00006247 | 0.00004368 | 122.638889 |
| 62 | 0.00006263 | 0.00004365 | 120.875323 |
| 6.35E+01 | 0.00006197 | 0.00004371 | 121.603359 |
| 6.50E+01 | 0.00006169 | 0.0000437 | 119.680392 |
| Mouse14 | data |  |  |
| Acq_time (min) | Optical Targeted | Optical untargeted | GBCA |
| 4.08E+00 | 0.0000339 | 0.00002428 | 130.843121 |
| 5.65E+00 | 0.00003194 | 0.00002337 | 148.116812 |
| 7.20E+00 | 0.00003124 | 0.00002261 | 151.626457 |
| 8.75E+00 | 0.00003057 | 0.0000223 | 153.632181 |
| 1.13E+01 | 0.00002939 | 0.00002183 | 155.793301 |
| 1.28E+01 | 0.00002873 | 0.00002151 | 165.518126 |
| 1.44E+01 | 0.0000282 | 0.00002137 | 158.876828 |
| 16 | 0.00002748 | 0.00002136 | 154.724812 |
| 2.55E+01 | 0.00002382 | 0.00002078 | 161.746886 |
| 2.71E+01 | 0.00002344 | 0.00002074 | 160.881647 |
| 2.98E+01 | 0.00002308 | 0.00002073 | 156.743481 |
| 3.15E+01 | 0.00002258 | 0.00002083 | 162.077168 |
| 3.31E+01 | 0.00002207 | 0.00002084 | 156.874073 |
| 3.48E+01 | 0.00002137 | 0.00002074 | 156.068476 |
| 3.64E+01 | 0.0000209 | 0.0000206 | 150.006572 |
| 3.92E+01 | 0.00002 | 0.00002056 | 150.679245 |
| 4.08E+01 | 0.00001943 | 0.00002054 | 146.442442 |
| 4.25E+01 | 0.00001885 | 0.0000204 | 142.634513 |
| 4.42E+01 | 0.00001844 | 0.00002038 | 138.453254 |
| 4.58E+01 | 0.00001793 | 0.0000204 | 139.152427 |
| 5.77E+01 | 0.00001588 | 0.00001995 | 123.875133 |
| 5.94E+01 | 0.00001538 | 0.00001991 | 120.084588 |
| 6.11E+01 | 0.00001506 | 0.00001986 | 118.814077 |
| 6.27E+01 | 0.00001487 | 0.00001974 | 115.005088 |
| Mouse15 | data |  |  |
| Acq_time (min) | Optical Targeted | Optical untargeted | GBCA |
| 4.37E+00 | 0.00002961 | 0.00002145 | 184.302022 |
| 6.03E+00 | 0.0000312 | 0.00002226 | 204.589545 |
| 7.68E+00 | 0.00003171 | 0.00002252 | 214.574682 |
| 9.45E+00 | 0.00003174 | 0.00002274 | 217.459326 |
| 1.21E+01 | 0.00003049 | 0.0000232 | 222.990562 |
| 1.37E+01 | 0.00003038 | 0.00002338 | 220.484195 |
| 1.54E+01 | 0.00002998 | 0.00002353 | 221.117154 |
| 3.08E+01 | 0.00002625 | 0.0000247 | 202.340709 |
| 3.24E+01 | 0.00002592 | 0.00002497 | 195.615131 |
| 3.41E+01 | 0.0000255 | 0.00002497 | 193.977828 |
| 3.57E+01 | 0.00002491 | 0.00002511 | 192.788614 |
| 3.75E+01 | 0.00002456 | 0.00002537 | 191.060909 |
| 4.02E+01 | 0.00002359 | 0.0000255 | 188.046142 |
| 4.19E+01 | 0.00002324 | 0.00002552 | 184.603446 |
| 4.36E+01 | 0.00002326 | 0.00002549 | 179.629513 |
| 4.52E+01 | 0.00002278 | 0.00002559 | 175.01588 |
| 4.71E+01 | 0.00002254 | 0.00002575 | 169.375281 |
| 5.92E+01 | 0.00002009 | 0.00002688 | 151.321498 |
| 6.09E+01 | 0.00001993 | 0.00002701 | 145.340824 |
| 6.26E+01 | 0.00001961 | 0.00002709 | 144.918502 |
| 6.42E+01 | 0.00001924 | 0.00002729 | 134.058577 |
| 6.59E+01 | 0.00001897 | 0.00002734 | 90.3057678 |
| Mouse16 | data |  |  |
| Acq_time (min) | Optical Targeted | Optical untargeted | GBCA |
| 4.67E+00 | 0.00002531 | 0.00001272 | 157.195827 |
| 6.37E+00 | 0.00002597 | 0.00001309 | 169.963884 |
| 8.08E+00 | 0.00002642 | 0.00001356 | 177.773274 |
| 9.78E+00 | 0.0000266 | 0.0000141 | 190.364366 |
| 1.25E+01 | 0.00002722 | 0.00001469 | 194.103896 |
| 1.42E+01 | 0.00002651 | 0.00001503 | 194.160915 |
| 1.59E+01 | 0.00002629 | 0.00001546 | 192.643616 |
| 2.59E+01 | 0.00002469 | 0.00001721 | 183.576645 |
| 2.76E+01 | 0.0000242 | 0.0000174 | 178.305377 |
| 2.93E+01 | 0.00002378 | 0.00001762 | 176.952648 |
| 3.20E+01 | 0.0000232 | 0.00001793 | 170.338283 |
| 3.37E+01 | 0.00002275 | 0.00001819 | 167.249599 |
| 3.54E+01 | 0.0000224 | 0.0000185 | 163.829856 |
| 3.71E+01 | 0.00002199 | 0.00001862 | 159.753247 |
| 3.89E+01 | 0.00002165 | 0.00001869 | 155.491573 |
| 4.16E+01 | 0.00002097 | 0.00001881 | 151.642857 |
| 4.33E+01 | 0.00002059 | 0.00001889 | 147.939005 |
| 45 | 0.00002018 | 0.00001896 | 141.235955 |
| 4.67E+01 | 0.00001975 | 0.00001907 | 133.172552 |
| 4.84E+01 | 0.00001932 | 0.00001917 | 134.239165 |
| 6.08E+01 | 0.0000168 | 0.00001993 | 107.930979 |
| 6.25E+01 | 0.00001652 | 0.00001998 | 103.989567 |
| Mouse17 | data |  |  |
| Acq_time (min) | Optical Targeted | Optical untargeted | GBCA |
| 4.08E+00 | 0.00003575 | 0.00003362 | 191.036013 |
| 5.58E+00 | 0.00003476 | 0.00003357 | 187.532634 |
| 7.08E+00 | 0.00003297 | 0.00003379 | 184.191011 |
| 8.58E+00 | 0.00003209 | 0.00003395 | 177.979833 |
| 11 | 0.0000294 | 0.00003456 | 164.412273 |
| 1.25E+01 | 0.00002739 | 0.00003435 | 151.934601 |
| 1.41E+01 | 0.00002567 | 0.00003416 | 143.222126 |
| 1.57E+01 | 0.00002421 | 0.00003424 | 140.454416 |
| 26 | 0.00001856 | 0.00003583 | 107.016398 |
| 2.85E+01 | 0.00001813 | 0.00003604 | 105.859273 |
| 3.01E+01 | 0.00001768 | 0.00003669 | 101.279666 |
| 3.17E+01 | 0.0000168 | 0.0000374 | 97.3655337 |
| 3.33E+01 | 0.00001707 | 0.00003748 | 93.2772809 |
| 3.50E+01 | 0.00001661 | 0.00003752 | 90.1538462 |
| 3.75E+01 | 0.00001592 | 0.00003765 | 83.6048266 |
| 3.91E+01 | 0.00001512 | 0.00003785 | 81.5128205 |
| 4.07E+01 | 0.00001483 | 0.00003794 | 79.2522361 |
| 4.23E+01 | 0.00001441 | 0.00003792 | 73.084675 |
| 4.39E+01 | 0.00001413 | 0.00003787 | 70.2710197 |
| 5.55E+01 | 0.00001226 | 0.00003796 | 55.4525939 |
| 5.72E+01 | 0.00001206 | 0.00003818 | 52.8002385 |
| 5.88E+01 | 0.00001172 | 0.00003834 | 50.86613 |
| 6.04E+01 | 0.00001127 | 0.0000384 | 50.1476569 |
| 6.20E+01 | 0.00001101 | 0.00003846 | 48.2519894 |
| Mouse18 | data |  |  |
| Acq_time(s) | Optical Targeted | Optical untargeted | GBCA |
| 3.78E+00 | 0.00006005 | 0.00004831 | 234.175602 |
| 5.10E+00 | 0.00005859 | 0.00004743 | 225.476726 |
| 6.45E+00 | 0.0000565 | 0.00004649 | 221.203531 |
| 7.80E+00 | 0.00005446 | 0.00004553 | 213.196469 |
| 1.02E+01 | 0.00005075 | 0.0000442 | 200.547078 |
| 1.17E+01 | 0.00004934 | 0.00004363 | 194.302087 |
| 1.32E+01 | 0.00004678 | 0.00004311 | 187.836918 |
| 1.47E+01 | 0.00004442 | 0.00004267 | 178.580738 |
| 1.62E+01 | 0.00004239 | 0.00004238 | 168.530672 |
| 2.70E+01 | 0.00003083 | 0.00004063 | 118.862921 |
| 2.85E+01 | 0.00002964 | 0.00004046 | 116.591653 |
| 30 | 0.00002879 | 0.00004018 | 111.158587 |
| 3.15E+01 | 0.00002789 | 0.00003981 | 105.302521 |
| 33 | 0.00002683 | 0.00003963 | 102.290365 |
| 3.55E+01 | 0.00002567 | 0.00003964 | 96.9868637 |
| 3.70E+01 | 0.00002494 | 0.00003965 | 94.9737815 |
| 3.85E+01 | 0.00002441 | 0.00003961 | 91.3418605 |
| 40 | 0.00002362 | 0.00003963 | 88.6119601 |
| 4.15E+01 | 0.00002288 | 0.00003962 | 85.1229236 |
| 5.25E+01 | 0.00001947 | 0.00003923 | 60.9521595 |
| 5.40E+01 | 0.00001912 | 0.00003922 | 58.4465116 |
| 5.55E+01 | 0.00001862 | 0.00003918 | 56.8574751 |
| 57 | 0.00001819 | 0.0000392 | 56.2425249 |
| 5.85E+01 | 0.00001838 | 0.00003939 | 54.5803987 |
| 6.11E+01 | 0.00001799 | 0.00003949 | 51.520598 |
